# Efficacy of warming needle moxibustion in the treatment of ankylosing spondylitis: A protocol of a randomized controlled trial

**DOI:** 10.1097/MD.0000000000025850

**Published:** 2021-05-21

**Authors:** Weizhong Ding, Shirong Chen, Xuexiang Shi, Yang Zhao

**Affiliations:** Department of Orthopedic Surgery, The Second Affiliated Hospital, Chongqing Medical University, Chongqing, China.

**Keywords:** ankylosing spondylitis, protocol, randomized controlled trial, warm needle moxibustion

## Abstract

**Background:**

Ankylosing spondylitis is a recurrent autoimmune disease, which has a high disability rate and seriously affects patients’ daily life. Conventional treatment cannot effectively solve the clinical problems of patients, and long-term medication is accompanied by adverse reactions. The evidence shows that warming needle moxibustion has advantages in the treatment of ankylosing spondylitis, but there is still a lack of clinical studies on warm acupuncture alone and long-term follow-up.

**Methods:**

This is a prospective randomized controlled trial to study the efficacy and safety of needle warming through moxibustion in the treatment of ankylosing spondylitis. It was approved by the Ethics Committee of Clinical Research of our hospital. Patients were randomly assigned to an observation group or a control group. The patients were followed up for 6 months after 30 days of treatment. Observation indicators include; activity index, functional ability, Bath Ankylosing Spondylitis Metrology Index, inflammatory indicators, adverse reactions, and so on. Finally, SPASS 22.0 software is used for statistical analysis of the data.

**Discussion:**

This study will evaluate the clinical efficacy of warming needle moxibustion in the treatment of ankylosing spondylitis. The results of this study will provide a reference basis for the clinical use of warm needle moxibustion in the treatment of ankylosing spondylitis.

**Trial registration:**

OSF Registration number: DOI 10.17605/OSF.IO/GWPX3

## 1 Introduction

Ankylosing spondylitis (AS) is a chronic autoimmune inflammatory disease that mainly damages the spine and sacroiliac joints, leading to progressive bone fusion of the spine,^[1]^ involvement of peripheral joints, attachment points of tendons and ligaments and other tissues.^[2,3]^ The prevalence rate of AS in China is about 0.3%,^[4]^ which mainly occurs in young and middle-aged men aged 20 to 30. According to statistics, the average AS patient needs to stop working after 15.6 years of illness, and most of the loss of function occurs within 10 years after the onset of the disease.^[5]^ Progression of ankylosing spondylitis varies, with about one-third of patients developing into severe disability. Back pain, spasticity, limitation of thoracic dilatation, and limited spinal activity often occur in the early stage of the disease, leading to occupational disability and increasing the economic burden of life.^[6]^

Current guidelines for the treatment of AS recommend the use of nonsteroidal anti- inflammatory drugs and tumor necrosis factor antagonist. Conventional synthetic disease-modifying anti-rheumatic drugs represented by sulfasalazine, methotrexate, and others are recommended for patients with peripheral joint involvement.^[7]^ However, the long-term use of AS may bring potential cardiovascular, gastrointestinal, and renal risks,^[8]^ at the same time, the high price of some drugs also brings heavy economic burden to patients. Therefore, it is particularly important to seek safe and reliable complementary replacement therapy.

Acupuncture and moxibustion are both traditional treatments originating from China and play an important role in the field of complementary and alternative therapies; it is widely used in the treatment of rheumatoid arthritis, osteoarthritis, low back pain, AS, and other diseases with reliable efficacy.^[9–13]^ Warm needle moxibustion is a kind of treatment combining moxibustion and acupuncture, the needle is inserted into the acupoint, and then the moxa stick is burned on the needle handle. The heat is introduced into the acupoint through the needle body, which has both acupuncture effect and warm effect.^[14]^ It has been clinically proved that Warm needle moxibustion can relieve pain, reduce inflammatory reaction, and assist in helping restore joint function.^[15]^ Although there have been studies on the treatment of AS with warm needle moxibustion, and positive conclusions have been drawn,^[16,17]^ most of them adopt the combined treatment scheme, there is a lack of randomized controlled studies on the single application of warm needle moxibustion in the treatment of ankylosing spondylitis, and there is also a lack of follow-up observation on its long-term efficacy. Therefore, we intend to evaluate the efficacy and safety of warm needle moxibustion alone in the treatment of AS through this randomized controlled trial.

## 2 Materials and methods

### 2.1 Study design

**Figure 1:**
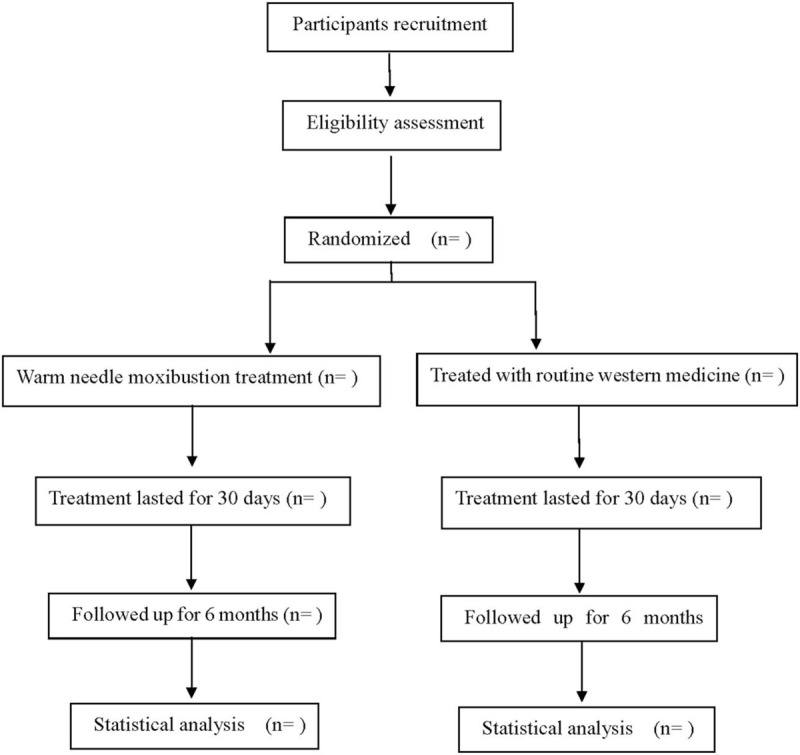
Flow diagram.

This is a prospective randomized controlled trial to study the efficacy and safety of warm acupuncture in the treatment of ankylosing spondylitis. This experiment will follow the reporting criteria of controlled trial intervention^[18]^ and comprehensive trial.^[19]^ The flow chart is shown in Figure 1.

### 2.2 Ethics and registration

This study protocol is in line with the Declaration of Helsinki and has been approved by the Clinical Research Ethics Committee of our hospital. This experiment has been registered at open science framework (registration number: DOI 10.17605/OSF.IO/GWPX3). Before randomization, all patients are required to sign an informed consent form, they could choose whether to continue the trial at any time.

### 2.3 Sample size

Sample size estimation is based on the mean and standard deviation of Bath Ankylosing Spondylitis Disease Activity Index (BASDAI)^[20]^ scores after treatment. According to the results of the pilot study, the observation group was 2.37 ± 1.52, the control group was 3.45 ± 1.46. Setα=0.025, unilateral test, *β*=0.10. The software PASS15.0 calculated that each group needed 42 participants, with an estimated drop-out rate of 20%, and 53 patients would be included in each group.

### 2.4 Patients

#### 2.4.1 Inclusion criteria

Consistent with the diagnosis of ankylosing spondylitis (diagnostic criteria refer to the Modified New York criteria for AS^[21]^); The age is 18 to 65 years old. BATH AS disease activity index (BASDAI)^[20]^ ≥4; People who have not used antirheumatic drugs to improve the disease have been used in the past 1 month, did not use biological agents in recent 3 months, and nonsteroidal anti-inflammatory drugs in the last 2 weeks; The patient who has strong compliance and signs the informed consent form.

#### 2.4.2 Exclusion criteria

Patients with major organ injuries or malignant tumors, infectious diseases or hematological diseases; People with other rheumatic diseases; Women in pregnancy or lactation; Evidence of active upper gastrointestinal ulcer within 1 month, or history of gastroduodenal perforation and upper gastrointestinal bleeding within 1 year; Allergic to drugs used in this study.

### 2.5 Randomization and blinding

Eligible participants were randomly assigned to either the treatment group or the control group in a 1:1 ratio using a central-network-based randomization tool. Random sequences are generated by independent statisticians who were not involved in the implementation of the experiment or statistical analysis using SAS 9.3 software. (SAS Institute, Cary, NC). The clinical research coordinator enters participant information on the tablet and gives the participant a random number. The research assistant got the assignment of participants from the computer. Throughout the study, the research assistant was responsible for screening, recruiting participants, and assigning random numbers to participants who had been included. Given the operational nature of the intervention, participants and operators may be aware of the random allocation. However, the assessors of the results of the study and the statisticians who are responsible for the statisticians of data statistics and analysis were not informed of the distribution.

### 2.6 Intervening measure

1. Observation group (warm needle moxibustion treatment); The patient was placed in the prone position, and the operation site was fully exposed. Use 75% alcohol topically to disinfect the acupuncture site, and the acupuncture points are: Huatuo jiájǐPoint(EX-B2), Bilateral shènshù point(B23), dàzhuī point(GV14), yāoyángguān point(GV3), Bilateral wěizhōng point(B40). Use disposable stainless-steel acupuncture needles (0.25 mm × 40 mm, Suzhou Hualun Medical Appliance Co, Ltd, Suzhou, China), and acupuncture is performed by an acupuncturist who is licensed in traditional Chinese medicine and has at least 3 years of clinical experience, After acupuncture is arrival of qi, add a section of moxa strips (Nanyang Baicaotang Co, Ltd, China, specifications: 18 mm × 27 mm) 27 mm long to the handle of the needle. The bottom of the moxa section is about 20 mm away from the skin, and is spaced with a small piece of paper. Ignite the bottom of the moxa section and finish the treatment after burning out the moxa stick, each treatment was 30 minutes, once every other day, and the treatment lasted for 30 days.
2. Control group (treated with routine western medicine): Indomethacin enteric-coated tablets (Datong Liqun Pharmaceutical Co, Ltd, National Drug Approval H14020511), 25 mg/time, twice /d orally; Salicylazosulfapyridine (Shanghai Zhongweizanwei Pharmaceutical Co, Ltd, National Drug Approval H31020450) 1 g/time, 2 times/d, orally. The treatment continued for 30 days.

### 2.7 Evaluation criteria and efficacy judgment

#### 2.7.1 Primary outcome

BASDAI ^[20]^; Functional Ability (measured using the Bath Ankylosing Spondylitis Functional Index)^[22]^

#### 2.7.2 Secondary outcomes

Ankylosing spondylitis metrology index ^[22]^; chest expansion; nocturnal spinal pain; laboratory indicators (including; erythrocyte sedimentation rate, C protein response, HLA-B27).

#### 2.7.3 Adverse reaction

Discomforts related to treatment occurred during treatment.

All the above observation indexes were collected on the day before and after treatment. All patients were followed up for 6 months, and data were collected according to the same criteria every month.

### 2.8 Data collection and management

One or 2 assistants are responsible for all data collection and recording in the whole process. Personal information about potential participants and registered participants will be collected, shared, and kept in a separate repository to protect confidentiality before, during, and after the trial. Access to the database will be limited to researchers within the research team.

### 2.9 Statistical analysis

The collected data were statistically analyzed by SPSS 22.0 software. Counting data were tested by *χ*^2^ test; mean ± standard deviation was used for measurement data ($\bar{x}$ ± S), Independent sample *t* test is used for normal distribution. The skewness distribution is tested by the Mann–Whitney *U* test. The difference was considered statistically significant when *P *< .05.

## 3 Discussion

At present, the pathogenesis of AS is still in the exploratory stage, and the existing evidence shows that it is closely related to genetic and environmental factors.^[3,23]^ The goal of treatment is to reduce the pain of patients, return to daily life and work as much as possible, and delay the injury of body structure.^[24]^ However, the current treatment scheme cannot effectively solve the above problems. Patients suffer not only from physical pain, but also from psychological pressure. It is estimated that one-third of AS patients are accompanied by symptoms of depression,^[25]^ so it is urgent to seek new complementary and alternative solutions.

Modern research has shown that acupuncture combined with warming effect can not only regulate the overall condition of the body, enhance the phagocytic function of cells, reduce nerve excitability, activate the activity of central acetylcholine function, accelerate the release of enkephalin synthesis, and increase the pain threshold,^[26]^ but also promote local circulation, accelerate the metabolism of inflammatory substances, reduce the level of inflammatory factors in patients, and improve the body's inflammatory response.^[27]^ It has been proven that warm needle moxibustion has the advantages of safety, effectiveness, and low price.^[28]^ Through this study, we will explore the clinical efficacy of warm needling moxibustion alone in the treatment of AS. The results of this study will provide a new plan for the treatment of AS, which is beneficial to patients and clinical decision makers.

There are also some shortcomings in this study: due to the treatment method, this study could not be double blindness, which may have some influence on the results of the study: At the same time, the research in the single may have regionalization of the population.

## Author contributions

**Conceptualization:** Weizhong Ding and Xuexiang Shi

**Data curation:** Weizhong Ding and Shirong Chen

**Formal analysis:** Xuexiang Shi

**Funding acquisition:** Shirong Chen

**Software:** Shirong Chen and Yang Zhao

**Supervision:** Xuexiang Shi and Yang Zhao

**Writing – original draft:** Weizhong Ding and Shirong Chen

**Writing – review & editing:** Weizhong Ding and Shirong Chen
